# iREAD: a tool for intron retention detection from RNA-seq data

**DOI:** 10.1186/s12864-020-6541-0

**Published:** 2020-02-06

**Authors:** Hong-Dong Li, Cory C. Funk, Nathan D. Price

**Affiliations:** 1 Center for Bioinformatics, School of Computer Science and Engineering, Central South University, Changsha, Hunan Province 410083 People’s Republic of China; 2 Institute for Systems Biology, Seattle, WA 98109 USA

**Keywords:** Intron retention, RNA-seq, iREAD, Gene expression

## Abstract

**Background:**

Intron retention (IR) has been traditionally overlooked as ‘noise’ and received negligible attention in the field of gene expression analysis. In recent years, IR has become an emerging field for interrogating transcriptomes because it has been recognized to carry out important biological functions such as gene expression regulation and it has been found to be associated with complex diseases such as cancers. However, methods for detecting IR today are limited. Thus, there is a need to develop novel methods to improve IR detection.

**Results:**

Here we present iREAD (intron REtention Analysis and Detector), a tool to detect IR events genome-wide from high-throughput RNA-seq data. The command line interface for iREAD is implemented in Python. iREAD takes as input a BAM file, representing the transcriptome, and a text file containing the intron coordinates of a genome. It then 1) counts all reads that overlap intron regions, 2) detects IR events by analyzing the features of reads such as depth and distribution patterns, and 3) outputs a list of retained introns into a tab-delimited text file. iREAD provides significant added value in detecting IR compared with output from IRFinder with a higher AUC on all datasets tested. Both methods showed low false positive rates and high false negative rates in different regimes, indicating that use together is generally beneficial. The output from iREAD can be directly used for further exploratory analysis such as differential intron expression and functional enrichment. The software is freely available at https://github.com/genemine/iread.

**Conclusion:**

Being complementary to existing tools, iREAD provides a new and generic tool to interrogate poly-A enriched transcriptomic data of intron regions. Intron retention analysis provides a complementary approach for understanding transcriptome.

## Background

Historically being considered as transcriptional noise or ‘junk’, intron retention (IR) has recently been shown to carry out important biological functions such as regulating gene expression that is coupled with nonsense mediated decay [1], producing novel isoforms [2] and targeting specific cell compartments [3]. It has thus been gaining recent interest, especially as it relates to a putative role in health and disease. Tumor-specific IRs were overexpressed in lung adenocarcinoma tumors [4], and IRs were mostly increased in an analysis across 16 cancers [5]. IR appears to be a widespread mechanism of tumor-suppressor inactivation based on integrative analysis of exome and RNA sequencing data [6]. IR was also found to regulate gene expression in biological processes such as normal granulocyte differentiation [7], CD4+ T cell activation [8], and terminal erythropoiesis [9, 10]. Functions of IR were systematically discussed in two recent reviews [11].

Next generation sequencing has resulted in a vast amount of RNA-seq data, which provides a rich resource for the detection of IR in combination with bioinformatics tools. However, to the best of our knowledge, only a few computational tools have been developed thus far—and these tools are either not freely available or have limitations. The methods described in [12] and [5] are not publicly available. IRcall and IR classifier are also not currently available, possibly due to website changes [13]. The recently developed KMA (Keep Me Around) is an efficient method for IR detection [14]. It involves transcript quantification from the command line followed by intron retention analysis in R, which brings some inconveniences due to switching software environments. Also, the flat distribution of retained reads, a common feature of IR, is not well identified in KMA. By analyzing the number of exon-exon junction reads and exon-intron junction reads, IRFinder detects retained introns by calculating a retention ratio that reflects the relative expression of transcripts with the retained intron to those that span the intron but does not have the intron retained [15]. As a promising approach, IRFinder also has one limitation that the distribution pattern of intronic reads is not well addressed [15]. Also, IRFinder considers introns that completely overlap of exons of other transcripts, resulting in the fact that the identified IR events may be ambiguous. For methods like rMATS [16, 17] and MISO [18], they can only analyze annotated introns in gene models and therefore can not be applied to profile all intron retention events that exist in samples. Another limitation of rMATS is that it focuses on determining the changes of PSI (percent spliced in) values between two groups of samples; those introns which are retained but not differentially expressed are not considered, therefore one cannot obtain holistic profiles of all retained introns by these methods. Therefore, developing new algorithms for detecting intron retention remains to be in great need.

In this work, we present iREAD (intron REtention Analysis and Detector), for the identification of IR from poly-A enriched RNA-seq data (single-end and paired-end). iREAD takes as input an existing BAM file and an annotation file that contains a list of introns that do not overlap any exons of any other splice isoforms or genes. For convenience, such introns are called ‘independent introns’. The features of iREAD are several folds. Firstly, the use of independent introns can help avoid the confounding of exons that overlap with introns, thus enabling unambiguous identification of intron retention. Methods like IRFinder include introns completely overlapping with exons of other transcripts and the resulting IR events may be ambiguous. Secondly, iREAD detects retained introns through analyzing both splice junction reads and intron expression level that considers all the intronic reads to increase confidence. However, methods like IRFinder only look at the percentage of expression of intron-retained RNA calculated from junction reads and do not consider expression level of introns. Thirdly, based on information theory, we proposed a novel entropy score to assess ‘how flat’ intronic reads are distributed across the whole intron region, a feature unique to iREAD. Using both simulated RNA-seq data and a deep-sequencing poly-A enriched mouse RNA-seq data, we tested the performance of iREAD and compared it with existing tools.

## Algorithm and Implementation

### Algorithm

**Fig. 1:**
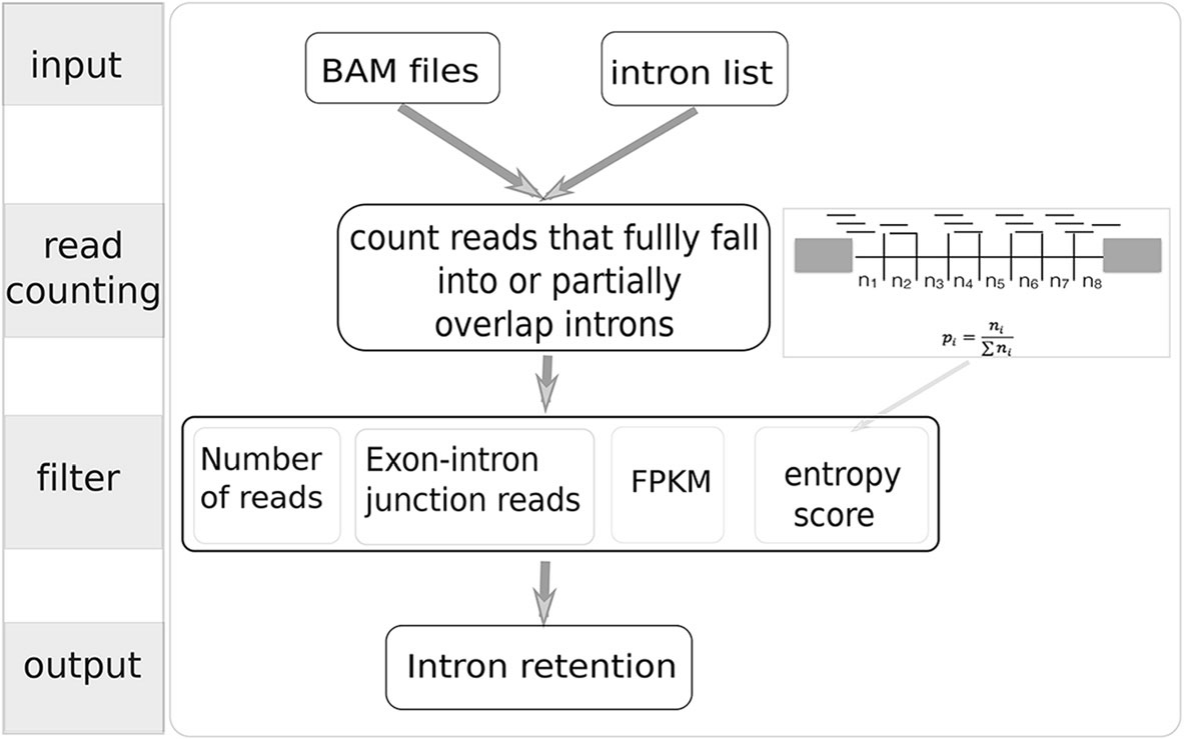
Schematic of the iREAD algorithm. iREAD takes two input files: a BAM file resulting from polyA-enriched RNA-seq read alignment and text file containing a list of independent introns that do not overlap with any exons of any transcripts. iREAD then counts the number of reads (partially) falling into the intron regions. After recording intronic reads, four filters (number of total reads, number of exon-intron junction reads, FPKM and entropy score) are calculated for filtering for high confidence intron retention events

iREAD takes two input files (Fig. 1): (1), a BAM file that is generated by aligning reads to a reference genome using tools such as STAR. The BAM file needs to be sorted by coordinates (the default of STAR) and to be indexed (can be done using the ‘samtools index’ command). (2), a text file containing the coordinates of independent introns that do not overlap with any exons of any other isoforms or genes. Independent introns are calculated by merging exons of all isoforms and genes of a given genome followed by subtracting them from spanning regions of genes using Bedtools [19]. Using ENSEMBL gene models (GTF format based on GRCh38), we identified independent introns together with their coordinates and parent-gene information for humans and mice, and provided them in the iREAD package. Pre-computed independent introns of humans and mice based on other versions of GTF annotation are made available at our website (https://github.com/genemine/iread). Independent introns of any other species can be identified in the same way. Since iREAD does not rely on known IR annotations, any retained introns (known or novel) are expected to be detected by iREAD.

Firstly, reads overlapping the independent intronic regions are extracted from BAM files using Samtools, and reads are counted with Bedops [20]. Since the resulting reads include spliced reads that can span but do not physically overlap introns, we developed a Perl script to count reads that fully reside in or partially overlap with the pre-calculated independent introns by considering both the read-spanning length (from the reference genome) and the coordinates of the independent introns. Specifically, iREAD counts and records the number of exon-intron junction reads, which provides direct evidence supporting retention of introns.

Because reads in retained introns are often flatly distributed across the whole intron region, we developed a score to characterize the ‘flatness’ of intronic reads based on information theory. We divide each intron into eight bins, count the number of reads in each bin, and record the number of reads in a vector, denoted as **r** = (n_1_, n_2_, …, n_8_). This vector is then converted to a probability mass distribution by normalization using the following formula:

1 $$ {p}_i=\frac{n_i}{\sum {n}_i},i=1,\dots, 8. $$

The entropy of this distribution is calculated as:

2 $$ entropy=-{\sum}_{i=1}^8{p}_i lo{g}_2\left({p}_i\right) $$

Because the maximal entropy for eight bins is 3, we divide the entropy by 3 and normalize it to be in the range [0,1], called the normalized entropy score (NE-score) for convenience.

3 $$ NE- score=\frac{entropy}{3} $$

Summing up, we recorded the number of total reads (denoted by T) and junction reads (denoted by J), and the NE-score of each intron. FPKM is also calculated for each intron to account for intron length. Based on these features, we can set threshold values to filter for IR events of high confidence. In the iREAD package, strict threshold values (T ≥ 20, J ≥ 1, FPKM≥3 and NE-score ≥ 0.9) are used as default to identify intron retention events conservatively and reliably. However, users can tune the default parameters in their own study.

iREAD is suitable for both single-end and paired-end sequencing data. For paired-end data, following the conventional practice in RNA-seq data analysis, the number of fragments is counted, i.e. a pair of reads is counted as one fragment.

### Implementation

We implemented the iREAD pipeline on the command line using a mixture of Perl and Python scripts. The command interface is implemented in Python using the ‘*argparse*’ module. iREAD requires a BAM file and a text file of independent introns as input. To determine IR events, we have implemented four filters: total reads, junction reads, FPKM and NE-score, which can be tuned easily by specifying the values of the optional parameters ‘-n’, ‘-j’, ‘-f’, and ‘-e’, respectively. To obtain high-confidence IR events, the number of total reads, junction reads, FPKM and NE-score is set to 20, 1, 3 and 0.9 respectively by default. Samtools and Bedops [20], which are two commonly used tools in NGS data analysis, need to be installed for running iREAD.

Using the Perl module Parallel::ForkManager, we recently parallelized iREAD so that it can use multi-cores, which makes the current version of iREAD (version 0.8.0) much faster than the previous one (version 0.6.0).

We implemented detailed and self-evident help document for iREAD. One can use the command ‘iread.py -h’ to obtain help information.

## Results

### User interface and usage

**Fig. 2:**
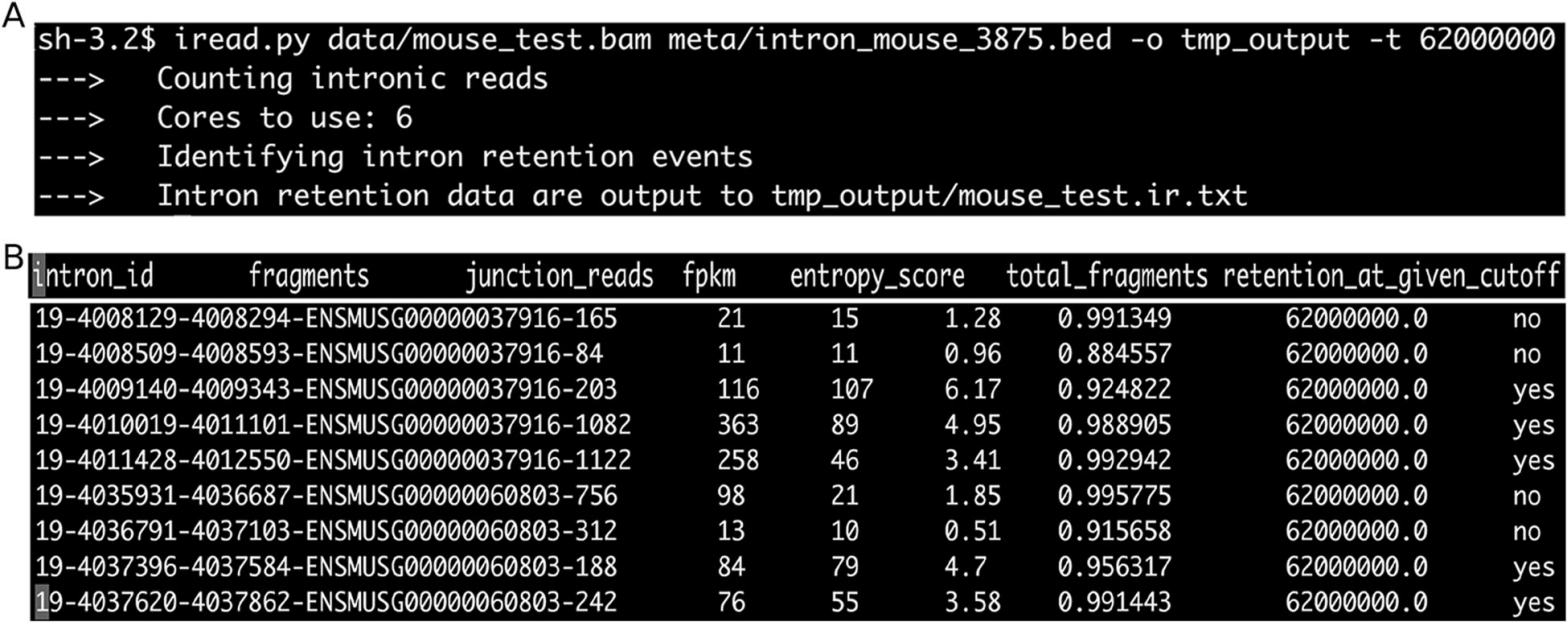
Illustration of the usage and output of iREAD. **a** an example run of iREAD on a mouse RNA-seq data that is included in the package. In addition to the RNA-seq BAM file and the intron file, ‘-o’ and ‘-t’ specify the output directory and the library size, respectively. **b** example rows of the output file resulting from (**a**). As shown, this file contains 7 columns. The first column is the intron ID. By default, iREADs uses strict criteria (fragments≥20, junction_reads≥1, FPKM≥3 and entropy_score > =0.9) to filter for intron retention events. The last column indicates whether an intron is retained (‘yes’) or not (‘no’). Note that the threshold for determining intron retention are optional parameters and can be tuned by the users

iREAD is implemented in Python with a command line interface, which can be run on Linux and Mac operating systems. For the convenience of testing, we have included an RNA-seq data (*data/mouse_test.bam*) and the annotation of independent introns of mice (*meta/intron_mouse_3875.bed*) in the iREAD folder. Using this data, users can run iREAD for IR detection using the command ‘*iread.py data/mouse_test.bam meta/intron_mouse_3875.bed -o tmp_output -t 62000000*’ (Fig. 2a), where ‘-o’ and ‘-t’ specify the output folder and the number of total fragments in the data.

After the iREAD run is finished, a file called mouse_test.ir is generated in the ‘tmp_output’ folder, where a list of introns together with their features such as the number of all reads (both junction and intronic), junction reads, FPKM and NE-score is stored (Fig. 2b). The last column of this file indicates whether the intron is retained (‘yes’) or not (‘no’) under default settings.

### Comparison of iREAD with IRFinder using simulated RNA-seq data

We compared the performance of iREAD to the state-of-the-art method IRFinder [15]. To the best of our knowledge, a gold standard dataset of experimentally validated intron retention events is not available for benchmarking. Therefore, we simulated an RNA-seq dataset using the BEER software [21]. As suggested by the current author of BEER, the option for generating novel splice isoforms is turned off, which is suitable for intron retention analysis. Based on the mouse ENSEMBL gene build GTF file (version 77) and using default setting, we generated a dataset of 30 million (30 M) paired reads. From this dataset, we randomly sampled half of the read pairs to generate a lower-depth dataset of 15 million (15 M) reads. For both datasets, based on the coordinates of each read, we first mapped the reads to all introns and then constructed three gold standard (GS) sets of IRs of different confidence level: GS1, GS2 and GS2, for which the minimum allowed FPKM of retained introns is set to 0.1, 0.3 and 0.5, respectively. GS3 collects IRs of the highest confidence, while the IRs in GS1 is relatively the least strictly-defined. GS2 includes IRs of the medium-level confidence. In addition, all IRs in these three sets have at least 10 paired end reads and at least one junction read that spans the exon-intron junction.

We ran iREAD and IRFinder on both the 15 M and 30 M datasets. The same gene annotation model (ENSEMBL GTF file, version 77) was used for iREAD and IRFinder. For iREAD, default settings of the four filters (Fig. 1) were used to determine IR events. For IRFinder, it mainly uses one metric, called IRratio ranging from 0 to 1, to describe to which extent an IR is retained, without providing an indicator of whether introns are retained or not.

First, we compared the performance of iREAD and IRFinder using their default settings to identify IR events. For IRFinder, the threshold of IRratio was set to 0.1 following the original paper [15]. Also, introns flagged with warning messages such as ‘low coverage’ by IRFinder were removed because of their low confidence. Default settings for iREAD are described in Section 2.2. For a given RNA-seq dataset, assuming that iREAD detects N_1_ retention events and IRFinder detects N_2_ introns with IRratio≥0.1, we sorted the retained introns by FPKM for iREAD and by IRratio for IRFinder, and chose the top *N* = min(N_1_,N_2_) to compare.

**Fig. 3:**
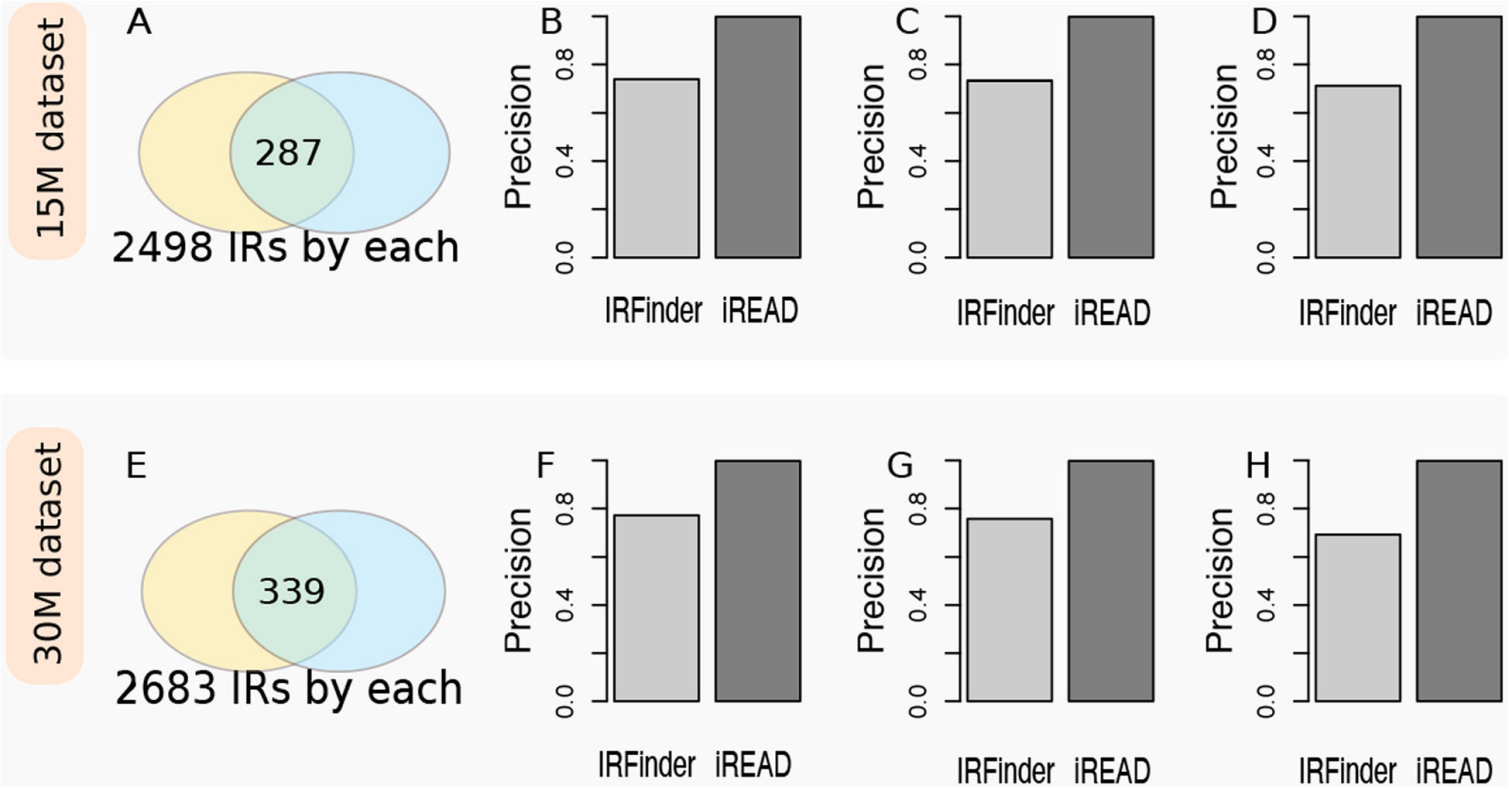
Comparison of the precision of iREAD with IRFinder on their identified intron retention events at default settings using simulated RNA-seq data with known ground-truth intron retention events. Two datasets were used, which are, for convenience, called15M dataset (containing 15 M reads) and 30 M dataset (containing 30 M reads). Firstly, retained introns were detected using both IRFinder and iREAD with default thresholds. Suppose N_1_ and N_2_ IR events were detected by each method, and the top ranked *N* = min(N_1_, N_2_) IR events of each method were compared in terms of the number of shared IR events and precision. N was found to be 2498 and 2683 for the 15 M (upper panel) and 30 M (lower panel) dataset, respectively. The sharing of identified IRs on the 15 M and 30 M dataset is shown in (**a**) and (**e**), respectively. On the 15 M dataset, the precision of IRFinder and iREAD based on the GS1 (FPKM≥0.1), GS2 (FPKM≥0.3) and GS3 (FPKM≥0.5) gold standard is presented in (**b**), (**c**) and (**d**), respectively. On the 30 M dataset, the precision of IRFinder and iREAD based on the GS1, GS2 and GS3 gold standard is presented in (**f**), (**g**) and (**h**), respectively. The results showed that iREAD achieved consistently higher precision than IRFinder on the IRs identified at default setting

The comparison of iREAD and IRFinder on the two simulated datasets are shown in Fig. 3. On the 15 M simulated data, the top ranked *N* = 2498 IR events by iREAD and IRFinder were compared. Out of the top ranked 2498 IR events, we found that 287 (11.5%) were shared by both methods (Fig. 3a), which indicates that the intron annotation and/or criteria used by iREAD and IRFinder capture different features of intron retention events. We further evaluated the precision (the number of true positives divided by the sum of true positives and false positives) and recall (the number of detected positives divided by the total number of positives in the dataset), for each method using the gold standard. We found that the precision of IRFinder on the GS1 (FPKM≥0.1), GS2 (FPKM≥0.3) and GS3 (FPKM≥0.5) gold standard were 0.73 (recall = 0.06), 0.73 (recall = 0.07) and 0.71 (recall = 0.08), respectively (Fig. 3b, c and d). The precision of iREAD was 0.99 in all the three gold standard sets, with recall equal to 0.08, 0.09 and 0.11 on GS1, GS2 and GS3, respectively (Fig. 3a). The reason why iREAD achieves higher precision may be that it directly counts the reads fully within introns and across exon-intron boundaries and therefore retained introns are expected to be found, as long as they are covered by a sufficient number of reads. In contrast, IRFinder uses IRratio, a more sophisticated criterion that measures the percentage of intron-retained transcripts; therefore, introns with high absolute expression but low relative expression may not be detected. Moreover, we looked into the top ranked IR events by IRFinder, and found that 36 out of the top 100 IR events completely overlap with exons of splice isoforms of the same gene. This result reflects that a proportion of IR events by IRFinder might be false positives because they overlap with exons; in contrast, the results of iREAD are confident because it only analyzes independent introns which by definition do not overlap with any exons of any genes. This is another reason why the sharing between IRFinder and iREAD is low and why IRfinder is less accurate. Nonetheless, the above result shows that most of IR events identified by both IRFinder and iREAD are accurate. On the 30 M simulated data, we detected slightly more intron retention events (Fig. 3e), likely due to the increased depth of the data. The precision comparison of IREAD and IRFinder is shown in Fig. 3f, g and h, respectively. The results on the 30 M dataset is similar to that on the 15 M dataset (Fig. 3).

**Fig. 4:**
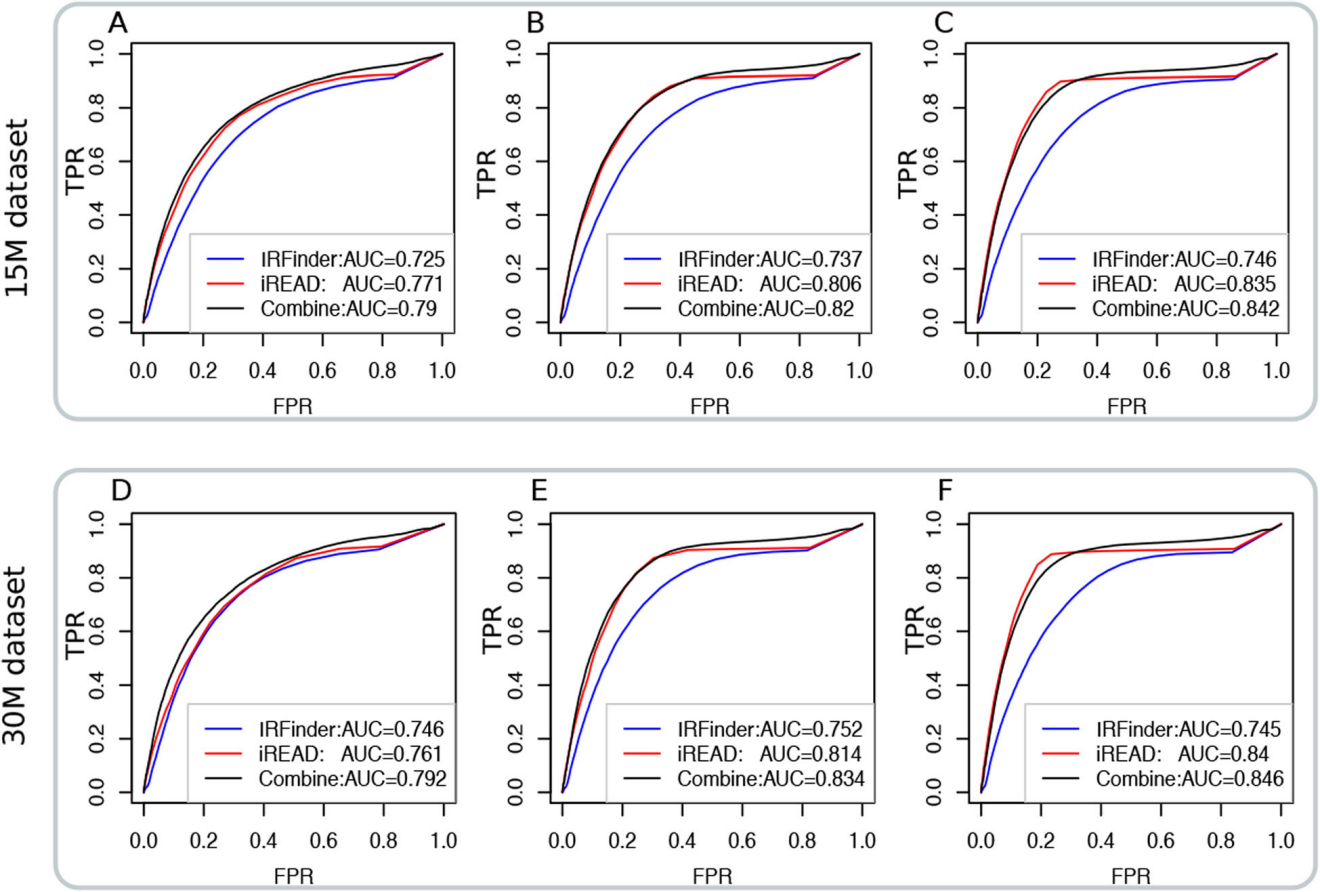
Performance comparison among iREAD, IRFinder and their combined score on the simulated 15 M and 30 M RNA-seq datasets with known ground-truth intron retention events in terms of AUC. The ROC curves of both methods based on the GS1(FPKM≥0.1), GS2 (FPKM≥0.3) and GS3(FPKM≥0.5) gold standard for the 15 M dataset is shown in (**a**), (**b**) and (**c**), respectively. Similarly, the ROC curves of both methods based on the GS1, GS2 and GS3 gold standard for the 30 M dataset are shown in (**d**), (**e**) and (**f**), respectively

Second, we compared iREAD with IRFinder in a comprehensive manner. Without using the default thresholds for detecting IRs, we pulled out all the introns analyzed by iREAD and IRFinder, and sorted them by FPKM and IRratio, respectively. We then calculated the ROC curves using the GS1 (FPKM≥0.1), GS2 (FPKM≥0.3) and GS3 (FPKM≥0.5) gold standard, respectively. The results on the 15 M and 30 M dataset based on GS1, GS2 and GS3 are shown in Fig. 4. For the 15 M dataset, we found that the AUC of iREAD on GS1, GS2 and GS3 are 0.77, 0.81 and 0.84, which is consistently higher than that of IRFinder whose AUC are 0.73, 0.74 and 0.75. Another observation is that, with increased confidence of gold standard from GS1 to GS3, the AUCs of both iREAD and IRFinder increase, being consistent with our expectation. The above observations hold on the 30 M dataset.

As iREAD complements IRFinder to some extent, we tested whether an ensemble of these two approaches could improve the results for IR identification. The scores used for evaluating intron retention propensity are intron expression level for iREAD and IRratio for IRFinder. One difficulty that faces us is that these two types of scores are not comparable. We therefore considered normalizing the scores. First, as the distribution of both scores of these two methods are heavily tailed, log_2_-transformation was applied to make the scores approximately normally distributed. To make the scores of iREAD and IRFinder in the same scale and comparable, the log_2_-transformed scores are further standardized to have zero mean and unit variance (with mean subtracted and divided by standard deviation using the *scale* function in R base package version 3.5.2). Let S_1_ and S_2_ be the log_2_-transformed and standardized score of iREAD and IRFinder. We then calculated their integrated score as $S=\frac{1}{2}\left({S}_1+{S}_2\right)$. The performance of this integrated score in terms of AUC was shown in Fig. 4. We observed that, for both the 15 M and 30 M datasets, the integrated score achieved better results than both iREAD and IRFinder, suggesting that these two approaches are complementary.

**Fig. 5:**
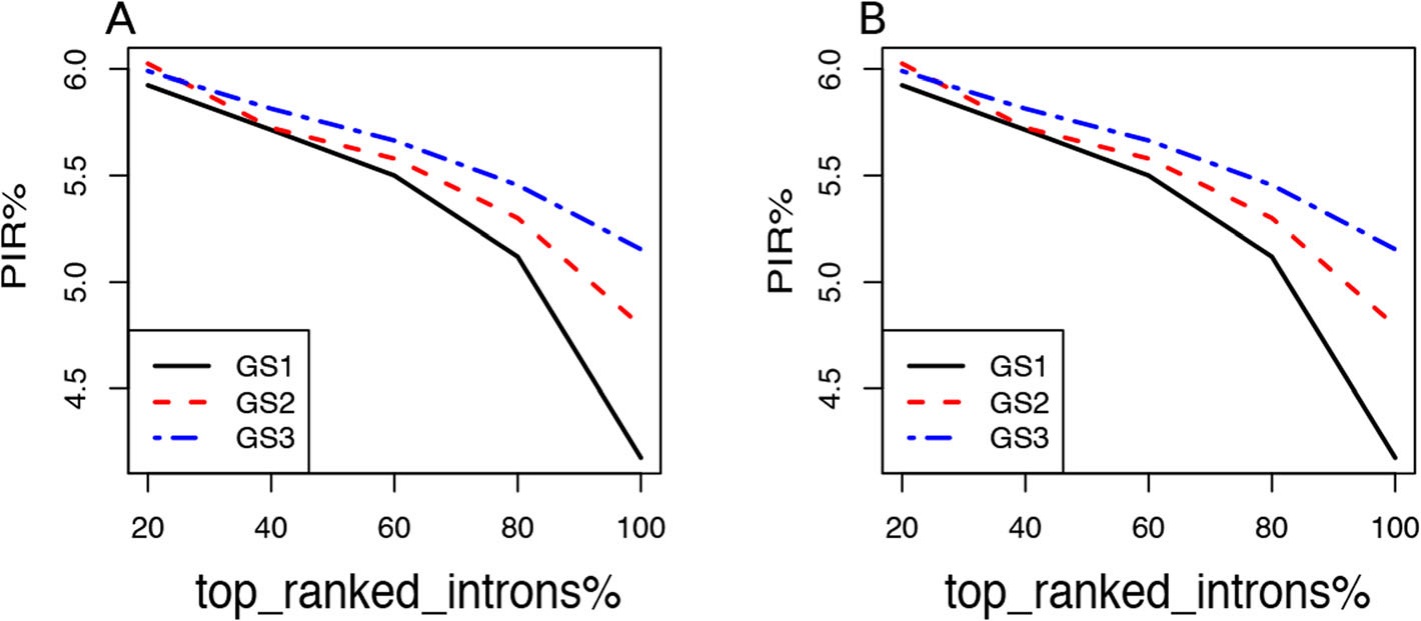
Performance improvement ratio (PIR) by the integrated score over the average performance of iREAD and IRFinder in terms of AUC. PIR was calculated for both the 15 M (**a**) and 30 M (**b**) datasets using the three different gold standard set of retained introns: GS1(FPKM≥0.1), GS2 (FPKM≥0.3) and GS3(FPKM≥0.5). PIR decreases as a function of increased numbers of positive retained introns, suggesting that iREAD and IRFinder complement most each other for the top ranked introns

We further showed that the more confident the retained introns are, the more improvement of AUC the integrated score would achieve. For each of the six data settings in Fig. 4, we ranked the positive retained introns by their expression level (FPKM). Then, we constructed a series of gold standard by including only a subset of the top ranked positives and all the negatives. Denote the proportion of selected top positives by R. We tested five values of R, which are 20, 40, 60, 80 and 100%. Note that *R* = 100% is equal to using all positive introns as in Fig. 4. For each *R* value, we calculated AUC for IRFinder, iREAD and the integrated score, respectively. Then we calculated the Performance Improvement Ratio (PIR) as $PIR=\frac{AUC_{integrated}-{AUC}_{mean}}{AUC_{mean}}$, where *AUC*_*integrated*_ represents the AUC of the integrated score and *AUC*_*mean*_ stands for the mean AUC of IRFinder and iREAD. The results of PIR for both the 15 M and 30 M datasets are presented in Fig. 5. It was observed that PIR decreases as a function of increased numbers of positive retained introns, suggesting that iREAD and IRFinder complement most each other for the top ranked introns and that their complementarity decreases for the lower ranked introns. Further, at a given value of *R* (e.g. 60%), we found that the lowest, medium and highest PIR values are achieved on the least confident (GS1), intermediate confident (GS2) and most confident (GS3) gold standard, respectively, again supporting that the highest gain in performance could be achieved for the most confidently retained introns.

Using simulated data with different sequencing depth and gold standard of retained introns of different quality, we found that both iREAD and IRFinder are accurate, and that many of their detected retention events are not shared due presumably to their differences in intron annotation and in the criteria for retention evaluation. We also showed that these two methods complement each other and using the integrated score of these methods could help improve the power for intron retention detection.

### Comparison of iREAD with IRFinder on mouse RNA-seq data

We further compared iREAD and IRFinder using a mouse brain RNA-seq data (available at https://www.synapse.org/#!Synapse: syn4486837, sample ID:256520).

This data was generated by polyA-enrichment and sequenced with 133 million paired end reads. Using the same approach for extracting the top ranked retained introns as used for the simulation experiments, we obtained 3238 IR events from each method, out of them 532 are shared.

**Fig. 6:**
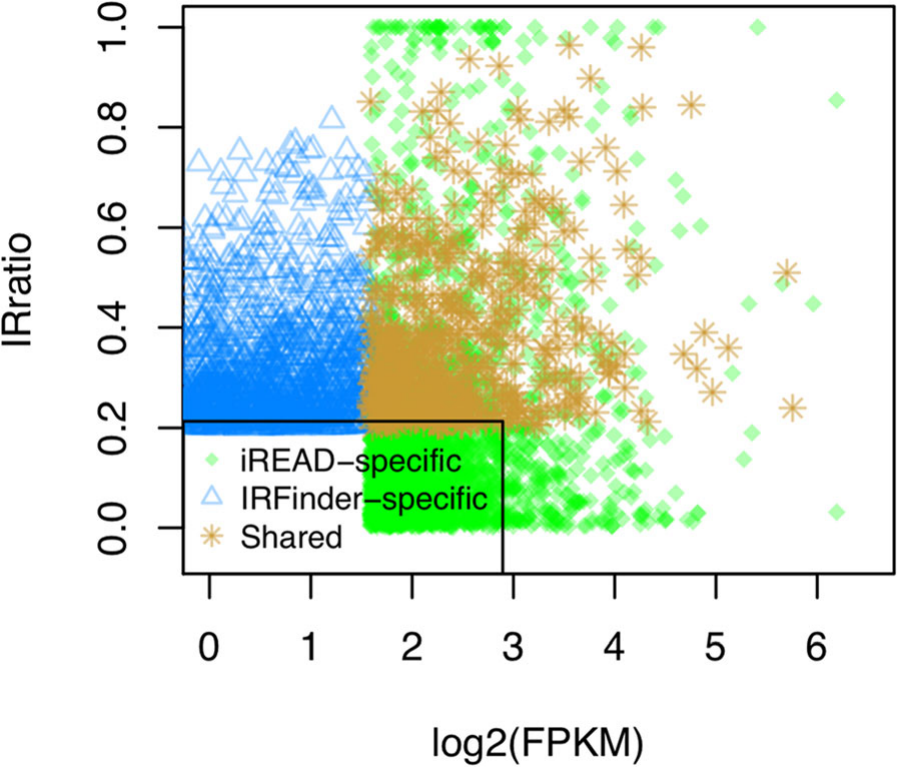
Systematic analysis of IRs detected by only IRFinder (IRFinder-specific) or iREAD (iREAD-specific) in the mouse data. We matched IRFinder-specific IRs to the introns used in iREAD and obtained their expression values in terms of FPKM; specifically, the IRFinder-specific introns overlapping with exons are not considered because iREAD does not consider them. We also matched iREAD-specific IRs to the introns used in IRFinder and obtained their IRratio values. IRFinder-specific IRs (with relatively higher IRratio) show generally lower expression than the retained introns detected by iREAD at default settings. iREAD-specific IRs (with relatively higher expression) show generally lower IRratio values than the retained introns detected by IRFinder at default settings. The shared IRs of the two methods have high IRratio and high expression

As the number of IRs shared by the two methods is small, we investigated why the two methods are disparate in their annotation by performing a systematic comparison of the 3238 IRs detected by each method. First, we excluded the 532 shared IRs detected by both methods and focused the analysis on the IRs specific to each method. For the 2706 IR events that are specific to IRFinder, only 1434 (53.0%) are annotated with ‘clean’ by the IRFinder software, which are IRs of high confidence; in contrast, a much higher percentage (69.2%) are annotated with ‘clean’ among the IRs shared by both methods. 741 (27.4%) are annotated with ‘known-exon’, indicating that a significant proportion of retained introns overlap with exons of other isoforms/genes; none of the IRs shared by both methods are annotated with ‘known-exon. Such introns cannot be detected by iREAD because it is designed to detect only independent introns. 386 (14.3%) are annotated with ‘anti-near’ (having a nearby gene on the antisense strand), which are another type of high quality annotation; this percentage is 30.0% for the IRs agreed by both methods. 145 (5.3%) are annotated with ‘anti-over’ (overlapping with another gene on the antisense strand), meaning that the reads in such introns may be from the anti-sense gene; the shared IRs have a lower percentage (0.8%) for this type of annotation. Further, we examined systematically why IRFinder-specific IRs are not detected by iREAD at default setting. We matched them to the independent introns used by iREAD and extracted their expression values in terms of FPKM. By comparison, it is found that IRFinder-specific IRs (not detected by iREAD) show generally lower expression than those retained introns detected by iREAD at default settings (Fig. 6). This observation suggests that iREAD may fail when the intron have low absolute expression (FPKM) though the relative expression of intron-retained transcripts (i.e. IRratio) can be high. For iREAD-specific IRs, we examined why they are not detected by IRFinder at default setting. We matched them to the introns used by IRFinder and extracted their IRratio values. It is found that iREAD-specific IRs (not detected by IRFinder) show generally lower IRratio values than those retained introns detected by IRFinder at default settings (Fig. 6). This observation suggests that IRFinder may fail when the relative expression of intron-retained transcripts is low though the intron may have high expression. This comparison again supports the complementarity between the two methods, and combining both methods may provide a more comprehensive set of IR events.

**Fig. 7:**
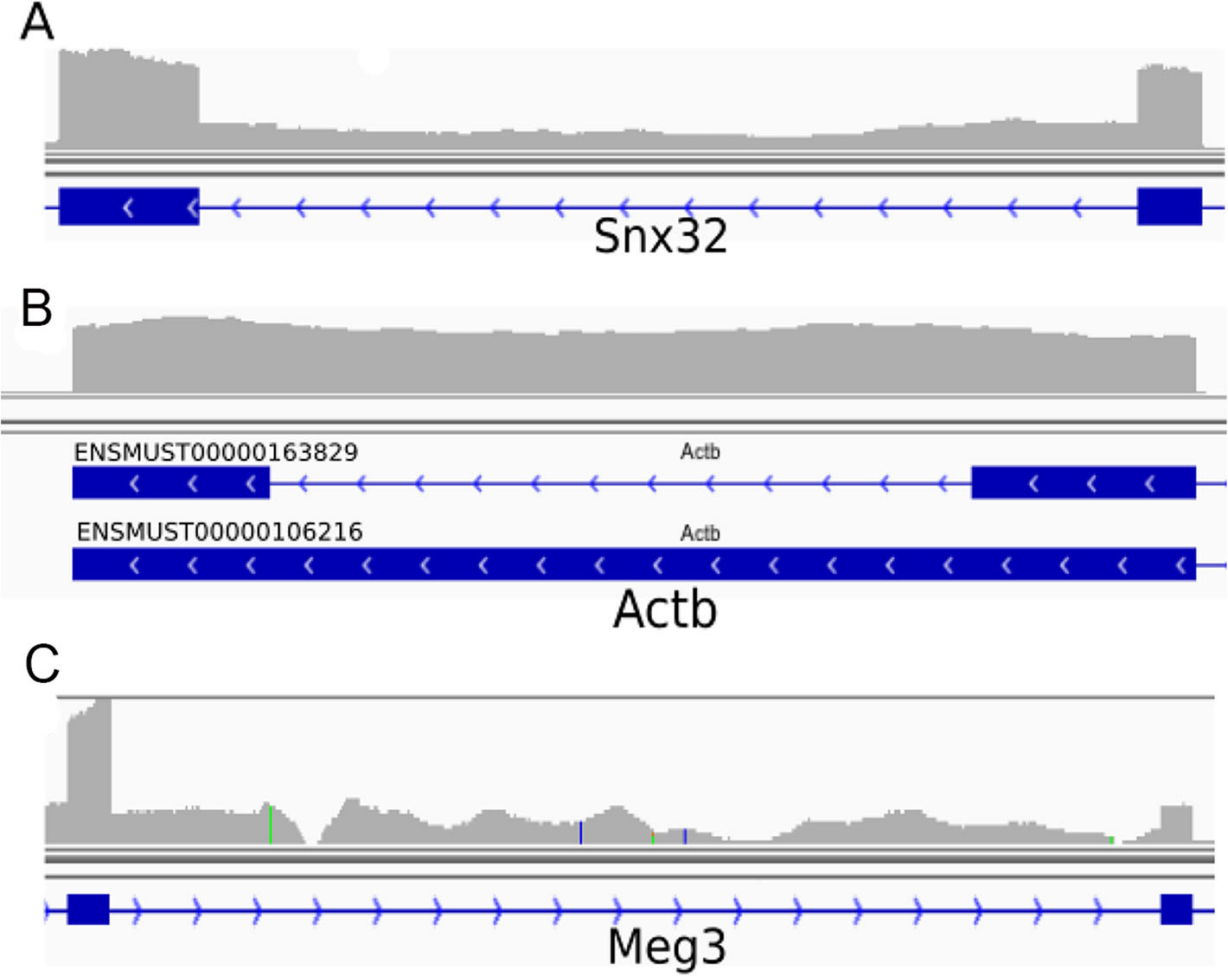
Examples of retained introns in the mouse RNA-seq data visualized in IGV (v2.3.90). **a** a retained intron (chr19:5496402–5,496,985) of *Snx32* identified by both IREAD and IRFinder. **b**, a retained intron (5:142,905,278-142,905,428 of *Actb*) identified by IRFinder but not by iREAD. As can be seen from the gene model in (**c**), this intron completely overlap with exons of another transcript (ENSMUST00000106216) of the same gene, indicating that this ‘retained intron’ is ambiguous because it could result from the transcription of exons. iREAD does not consider this intron to be retained because it only analyzes independent introns, i.e. unambiguous introns that do not overlap with any exons of any transcripts. **c**, a retained intron (chr22:109,546,542-109,549,290 of Meg3) identified by iREAD but not by IRFinder

As illustrations, we showed one example for each of the three categories: shared IRs, IRFinder-specific IRs and iREAD-specific IRs, respectively. For the shared introns, an example is a confidently retained intron (chr19:5496402–5,496,985) of *Snx32* (ENSMUSG00000056185) with an NE-score of 0.995 and coverage by 420 reads of which 157 are junction reads (Fig. 7a). The IRratio of this intron by IRFinder is 0.29, which is significantly higher than the threshold 0.1. In Fig. 7b, we showed an intron (5:142,905,278-142,905,428 of the gene *Actb*) identified to be retained by IRFinder but not by iREAD. With a closer look at the gene model in Fig. 7b, it can be found that this intron of the transcript ENSMUST00000163829 completely overlap with exons of another transcript (ENSMUST00000106216) of the same gene, indicating that this ‘retained intron’ is ambiguous because it could result from the transcription of exons. This observation explains why iREAD does not consider this intron to be retained because it only detects the unambiguous independent introns that do not overlap with any exons of any transcripts. However, in IRFinder, ambiguous intron retention events are also included. In Fig. 7c, we showed one example intron (chr22:109,546,542-109,549,290 of the gene *Meg3*) which was detected to be retained by iREAD but not by IRFinder. Clearly, this intron does not overlap with exons of any other transcripts, and all intronic reads must come from retained introns. We are not clear why this intron is missed by IRFinder.

### iREAD detects biologically relevant IRs

**Fig. 8:**
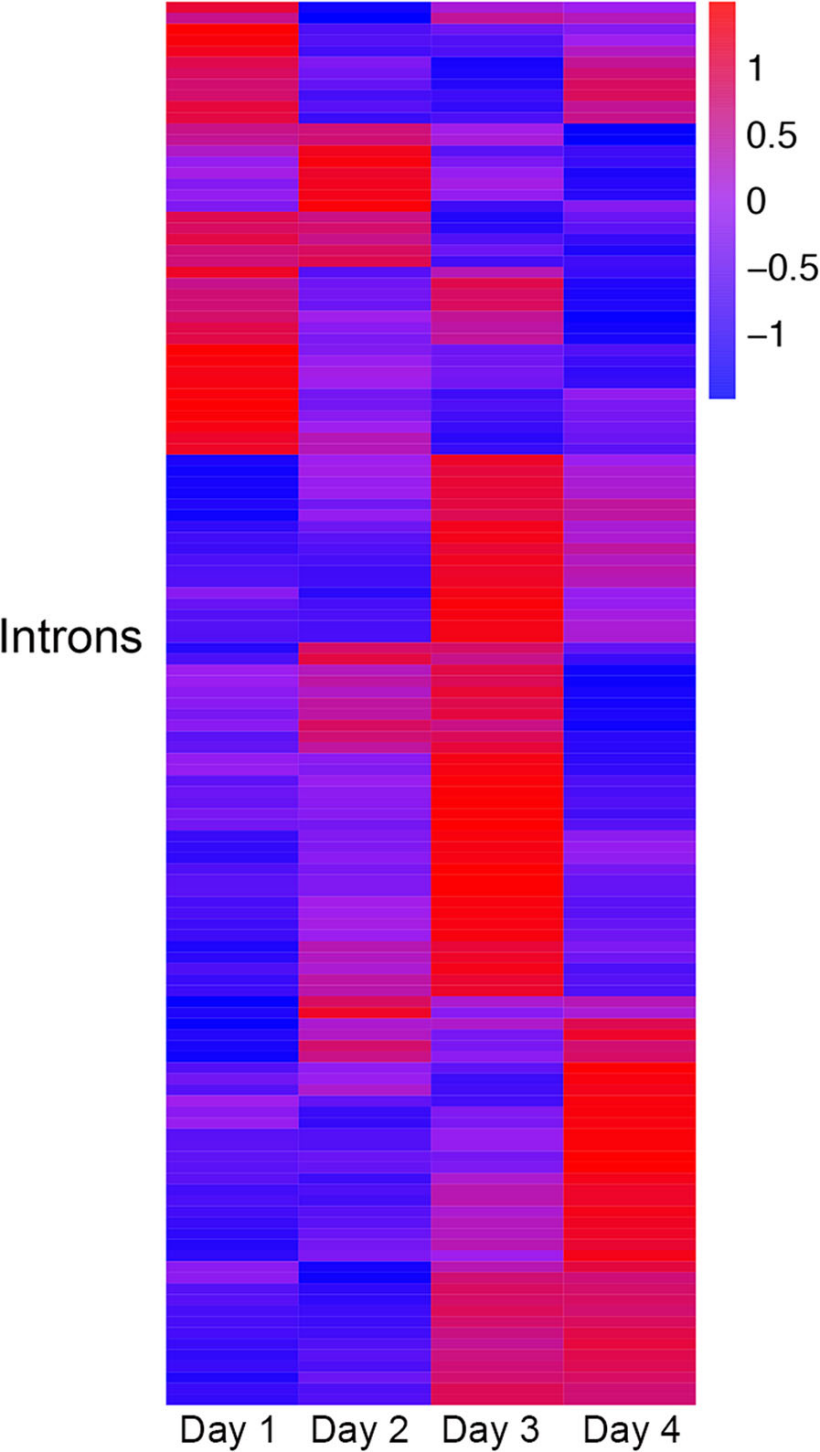
Heatmap of expression of differentially expressed retained introns between Day 1 and Day 4 in the human induced pluripotent stem cells (hiPSCs) dataset. Three samples are measured at each time point and the mean expression of the three samples are shown. Retained introns mark the developmental stages of hiPSCs during differentiation into neurons

To illustrate that iREAD is capable of detecting biologically meaningful IRs, we analyzed a dataset of human induced pluripotent stem cells (hiPSCs) that can be differentiated to neurons. This dataset contains samples at four time points (Day 1, Day 2, Day 3, Day 4), with 3 samples at each time point. Thus, there are a total of 12 samples. We downloaded the raw RNA-sequencing data in FASTQ format of the 12 samples, and apply iREAD to each sample to detect IR events. Using edgeR, we detected differentially expressed retained introns between Day 1 and Day 4. A total of 75 differential introns (FDR < 0.05) were detected. As shown in Fig. 8, these retained introns mark the developmental stages of hiPSCs during differentiation into neurons.

To understand the functions of the differential IRs, we performed Gene Ontology (GO) enrichment analysis for the parent genes of these IRs using the clusterProfiler software (v3.12). We found that the genes are enriched in a total of 22 GO biological process terms, suggesting that the detected IRs are not random but are related to biological pathways. Moreover, we found that some of these enriched terms are related to the differentiation of hiPSC into neurons. For example, *regulation of cholesterol biosynthetic process* (GO:0045540) was shown to be relevant to the neural stem cell differentiation [22]. Modulation of chemical synaptic transmission (GO:0050804) have been reported to be associated with human iPSC-derived neurons [23]. RNA splicing (GO:0008380) was implicated in human iPSCs that undergo neuron differentiation [24]. These results imply that biologically meaningful IRs can be uncovered with iREAD.

### Speed

**Fig. 9:**
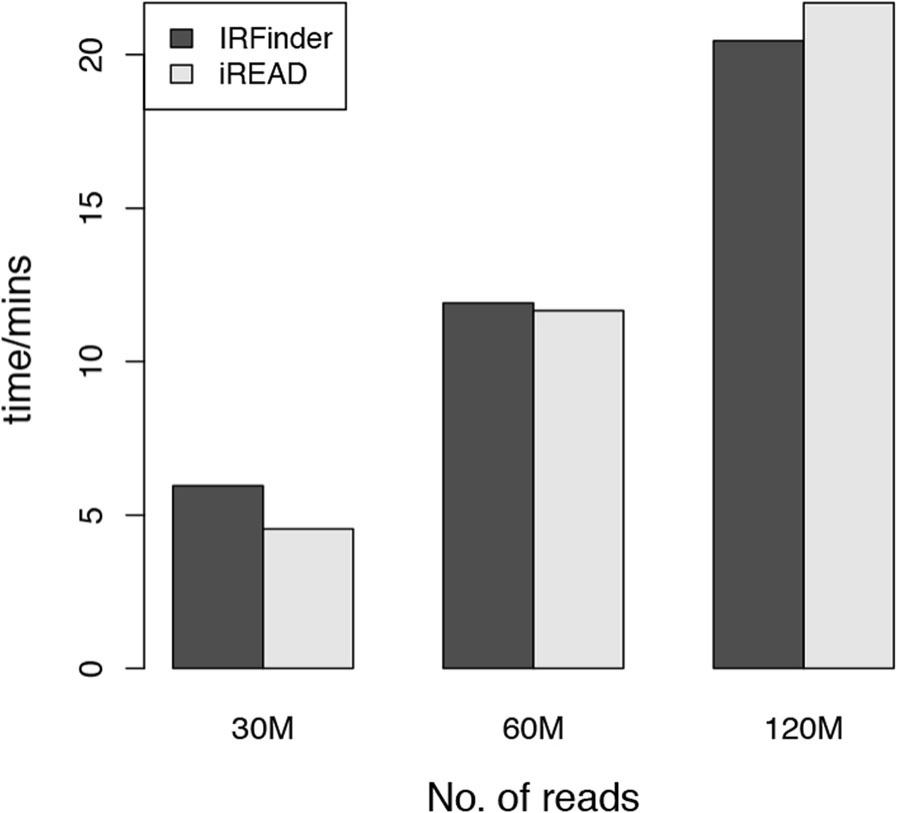
We compared the speed of iREAD to IRFinder using three data at different depth (30 M, 60 M and 120 M). The 30 M and 60 M data are sub-sampled from the original mouse RNA-seq data with 120 M reads. It shows that the speed of both methods are comparable

We compared the speed of IRFinder and iREAD for intron retention detection using the above-mentioned high sequencing depth mouse sample with 133 million reads (9.9G BAM file). From the raw BAM file, we also randomly sampled 30 and 60 million of the paired end reads to generate RNA-seq data of different sequencing depth for investigating how the speed of iREAD changes with the number of reads. On a commonly used server with 20 cores and 64G memory, iREAD takes only 5, 12 and 22 min for intron retention detection for the three sub-datasets at different depth (Fig. 9). Since most RNA-seq data have less than 60 million reads. iREAD is expected to finish IR event detection of one RNA-seq sample with ~ 10 min, which is efficient. We also tested IRFinder on the same computer, and found that the speed of both methods are comparable (Fig. 9).

## Conclusion

By quantifying intronic reads and mining their characteristics, we designed the iREAD algorithm for genome-wide detection of novel intron retention events from RNA-seq data. We implemented it as a command line software using Python. It works with both single-end and paired-end sequencing data that has been prepared using poly-A enrichment protocols. We compared iREAD with the recently published method IRFinder using simulated RNA-seq data with known ground truth intron retention events, and found that it achieves consistently higher accuracies than IRFinder and that iREAD complements IRFinder in terms of their criteria in detecting IR events. We showed that using the integrated score of both methods is a good way in practice because it can improve the performance of retained intron detection. Based on the simulation study, directly analyzing intronic reads as used in iREAD achieved higher sensitivity than using the IRratio metric in IRFinder. We found that iREAD is also efficient when tested on real data with very high sequencing depth (over 130 million reads). The resulting intron retention data can be further explored in various ways such as differential expression and functional enrichment, allowing for its use in many fields including the search for disease-associated gene expression signatures. We also illustrated that IRs identified by iREAD are biologically meaningful. Being complementary to existing tools, iREAD provides a new and generic tool to interrogate the previously largely neglected intronic regions from the angle of view of intron retention.

## Availability and requirements

- Project name: iREAD
- Project home: https://github.com/genemine/iread.
- Operating system(s): Linux, Mac
- Programming language: Python, Perl
- Other requirements: No
- License: No.
- Any restrictions to use by non-academics: No

## Data Availability

The source codes of iREAD and independent intron data are made freely available at: https://github.com/genemine/iread.

## Abbreviations

BAM: Binary version of sequence alignment map
FPKM: Fragments per kilobase of exon model per million mapped reads
IR: Intron retention
NE-score: Normalized entropy score
RNA-seq: RNA sequencing
